# Mechano-synthesized orange TiO_2_ shows significant photocatalysis under visible light

**DOI:** 10.1038/s41598-018-33772-6

**Published:** 2018-10-19

**Authors:** Ken-ichi Saitow, Yufeng Wang, Shintaro Takahashi

**Affiliations:** 1 Department of chemistry, Graduate School of Science, Hiroshima University, 1-3-1 Kagamiyama, Higashi Hiroshima, 739-8526 Japan; 2 Natural Science Center for Basic R&D (N-BARD), Hiroshima University, 1-3-1 Kagamiyama, Higashi Hiroshima, 739-8526 Japan

## Abstract

Nitrogen and carbon co-doped TiO_2_ particles with a brilliant yellow-orange color were produced mechanochemically by high-energy ball milling as one-pot synthesis. This facile synthesis required only grinding TiO_2_ with melamine at room temperature. Using monochoromatic lights with the same intensity in visible and UV, the photocatalytic activity of the TiO_2_ particles was accurately evaluated with respect to the degradation of an aqueous dye (methylene blue) solution. The activities under visible light (450 and 500 nm) were, respectively, 4 and 2 times higher than that of the unmilled TiO_2_ under UV light (377 nm), corresponding to 9 and 5 times higher than the UV under the solar light condition. The properties and structure of the co-doped TiO_2_ particles before and after milling were analyzed using eight experimental methods. As a result, it was found that the nitrogen replaced as an oxygen site in milled TiO_2_ has the highest concertation (2.3%) in the past studies and the structure of milled TiO_2_ is composed of a polymorphism of four different solid phases of TiO_2_, gives significant higher photocatalytic activity at visible light than that of UV light. A good repeatability of the photocatalyst was investigated by the number of cycles for the decomposition reaction of the aquesous dye solution.

## Introduction

Titanium dioxide (TiO_2_) is the most popular photocatalyst and is used for self-cleaning and self-sterilization applications, water and air purification, and as a water-splitting catalyst^1,2^. The TiO_2_ photocatalyst is activated by UV light due to its wide band gap (3.2 eV, λ = 387 nm). Visible-light activated TiO_2_ has attracted much attention^3–8^ because the solar light spectrum includes only 5% UV light and artificial room lighting also emits mainly visible light. Thus, metal-ion doping^4–7^, non-metal doping^3–8^, surface plasmon resonance of gold^9–11^ and Z-scheme process^12–14^ have been conducted for the synthesis of visible-light activated TiO_2_. However, almost all of these methods have required complex experimental conditions, such as ion-implantation facilities, vacuum conditions, and high-temperature synthesis over 400 °C for several hours. Another important issue for TiO_2_ photocatalysts is to compare the photocatalytic activity under visible light with that under UV light, and there have been many such reports to date^15–24^. However, there have been only a few systems that exhibit higher activity under visible light than that under UV light, such as N-doped TiO_2_ prepared by sputtering with N_2_ gas^15^, S-doped TiO_2_ prepared by annealing TiS_2_^16^ and Ag-TiO_2_ prepared by sol-gel and calcination processes^17^. Namely, the photocatalytic activities of these three systems under visible light were, respectively, 2.5^15^, 1.4^16^ and 2.2^17^ times higher than those under UV light. In addition, there have been no reports on colored TiO_2_ for the comparison of photocatalytic activities in UV and visible using monochromatic light with the same intensity. Therefore, the development of as many other systems as possible that exhibit higher photocatalytic activity under visible light than under UV light is crucial for chemical science and industrial applications. In addition, a facile synthesis method must be also essential for the commercialization of such photocatalysts.

As a sample preparation method, ball milling has attracted considerable attention as a physical synthesis method because a large number of particles can be easily obtained by simply grinding solid materials in a milling vessel with milling balls, i.e., one-pot facile synthesis at room temperature^25^. Thus, several studies on the production of TiO_2_ particles by ball milling have been reported^26–35^. The crystallite size, specific surface area, and crystal structure of TiO_2_ particles have been investigated as a function of the milling time^26,27^. The kinetics and mechanism of the phase transition were elucidated by either varying the TiO_2_ to ball weight ratio or by changing the milling ball and vessel material^28–30^. TiO_2_ particles that can absorb visible light have been synthesized by doping with nitrogen^31^, sulfur^32^, phosphorus^33^, and TiH_2_^34^. However, there have been only a few studies on the photocatalytic activity of TiO_2_^28,31,33,35^, and the effect of milling on the activity of TiO_2_ is not yet well understood, e.g., increases of photocatalytic activities^31,33^ and decrease of photocatalytic activity^28^. In our previous study, the photocatalytic activity of milled TiO_2_ was determined to be 136 times that of TiO_2_ before milling and 62 times that of the commercially available P25 catalyst by UV irradiation^35^. Such an extraordinary enhancement was attributed to the crystal phases that emerge at pressures as high as gigapascals during milling.

Here, nitrogen (N) and carbon (C) co-doped TiO_2_ particles were synthesized by high-energy ball milling as one-pot synthesis at room temperature. The activity under visible light was carefully compared with that under UV light under the same conditions. As a result, the activities of doped TiO_2_ under visible light at 450 and 500 nm were respectively 4 and 2 times higher than that of the unmilled pristine TiO_2_ under UV light (377 nm), corresponding to 9 and 5 times higher than the UV under the solar light condition. The TiO_2_ structure was composed of four polymorphisms, i.e. rutile, anatase, amorphous, and α-PbO_2_ phase, whose last one is referred to as either srilankite or TiO_2_-II phase is formed under high temperature and high pressure, ie. 600 °C, 5GPa^36^. The product obtained was a bright yellow-orange colored powder. The N and C concentrations in the TiO_2_ particles were highest as 2.3 and 1.3 wt%, respectively, in the previous studies.

**Figure 1:**
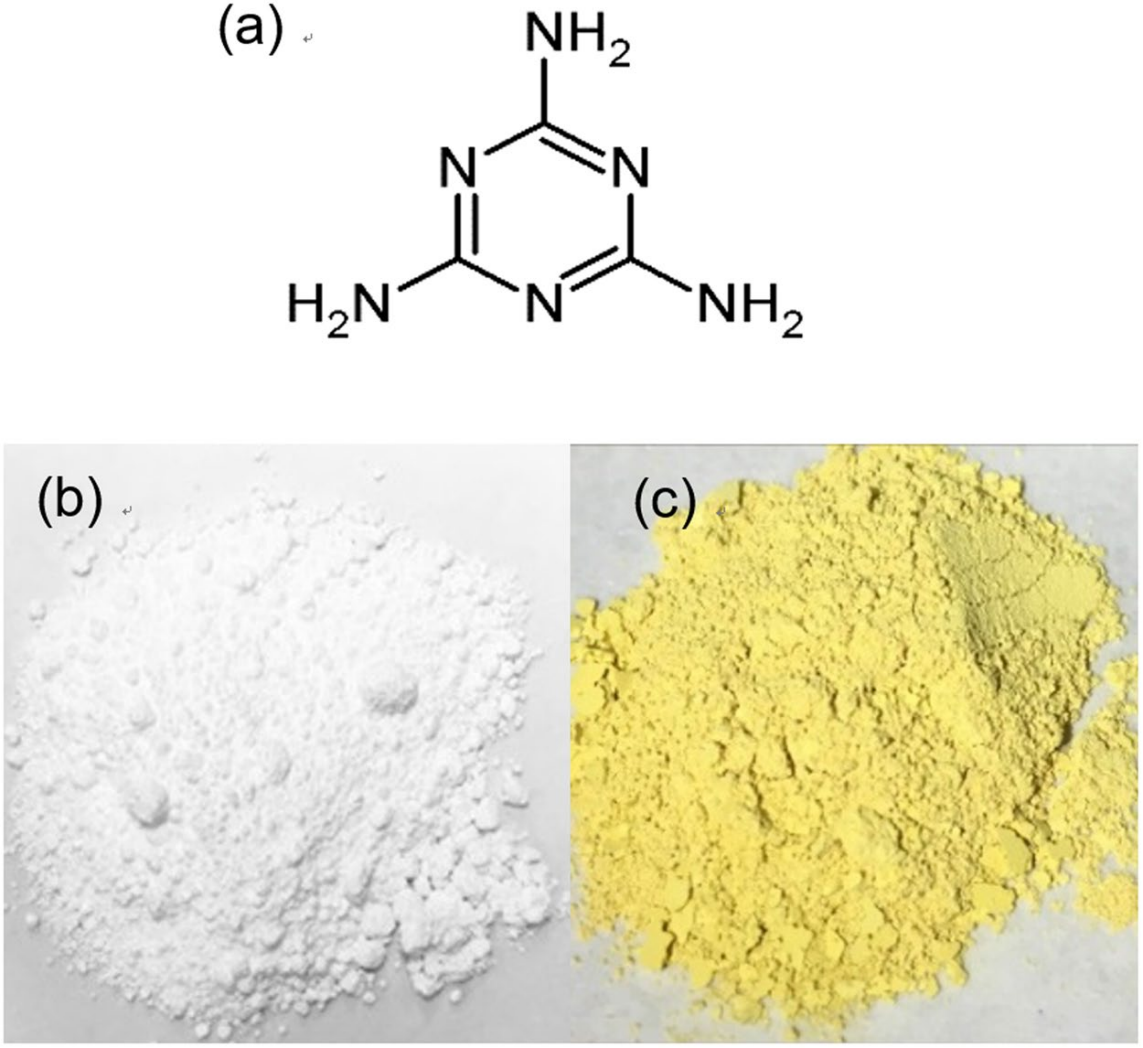
Sample materials and characterization. (**a**) Molecular structure of melamine. Photographs of (**b**) P25 (degussa) and (**c**) P25 milled with 5 wt% melamine.

A planetary ball mill was used to grind the samples^35,37,38^ (see Methods). The source used for the N and C atoms was melamine (C_3_H_6_N_6_, Fig. 1a), which has a large amount of N and C per molecule, 50 and 25 at%, respectively. A zirconium oxide (ZrO_2_) milling vessel and ZrO_2_ milling balls were used. The milling balls, TiO_2_ (Degussa, P25), and melamine powders were placed in the vessel, and milling was performed for various milling times (0–300 min), revolution speeds (0–600 rpm), and melamine concentrations (0–40 wt%). The photocatalytic activity was evaluated according to the photodegradation of an aqueous solution of methylene blue (MB) under visible and UV light. Namely, the TiO_2_ particles were placed in a quartz cuvette with MB aqueous solution and a stirring bar. Monochromated light (377, 450, or 500 nm) passed through a band-pass filter with a full width at half maximum (FWHM) of 10 nm was irradiated onto the cuvette at room temperature. UV-vis absorption spectra of the MB solution were recorded to evaluate the photocatalytic activity. These evaluations were accurately conducted using the same light intensity and the same TiO_2_ amounts. In addition, the TiO_2_ samples were characterized using 8 different experimental analyses, i.e. diffuse reflection spectroscopy, CHN elemental analysis, dynamic light scattering (DLS), transmission electron microscopy (TEM), high-resolution TEM (HR-TEM), X-ray diffraction (XRD), X-ray photoelectron spectroscopy (XPS), and surface area with the BET method. Thus, the relations between the photocatalytic activity and TiO_2_ structure were investigated before and after milling and discussed by comparing all the results.

**Figure 2:**
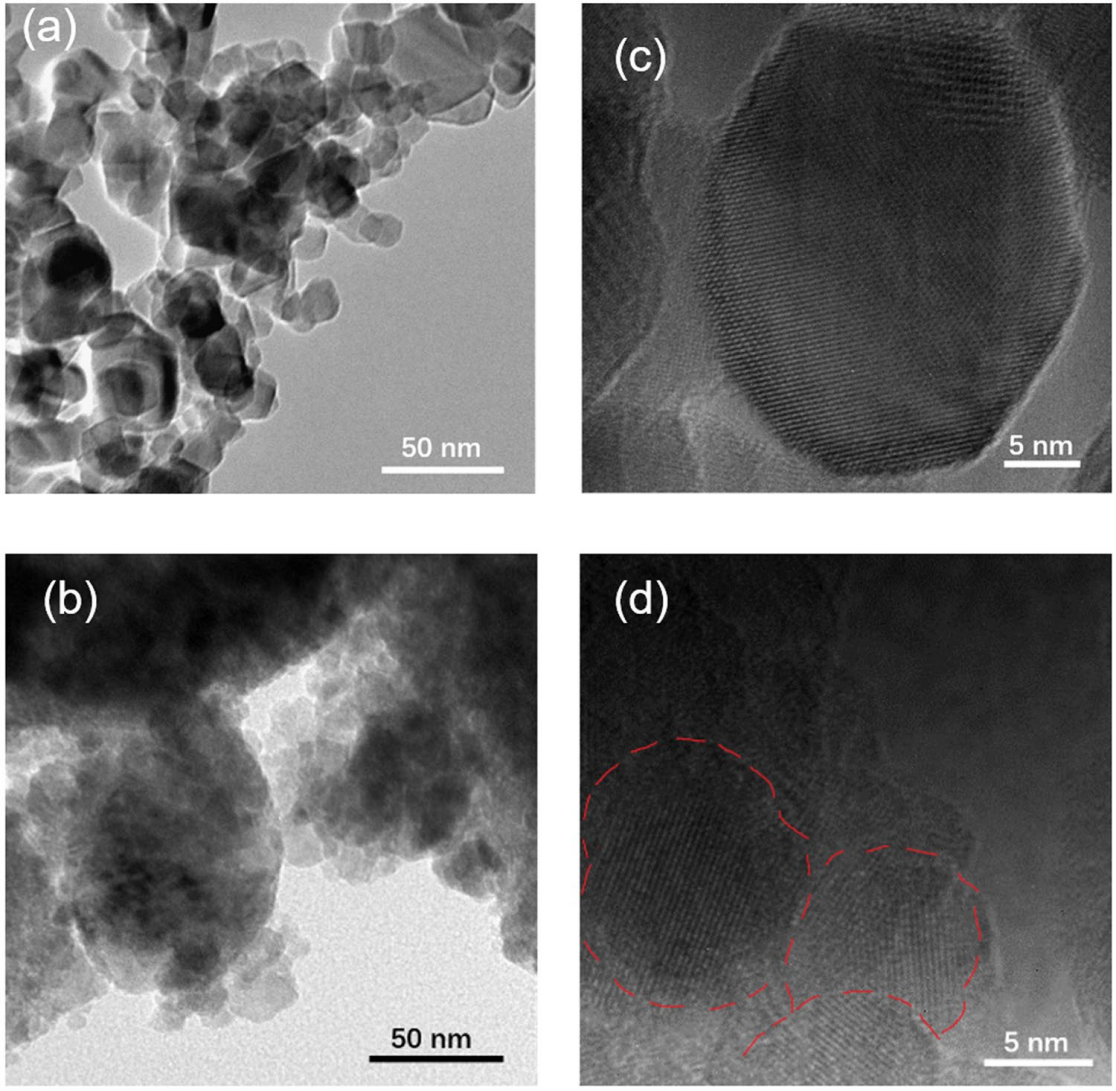
TEM images of (**a**) P25 before milling and (**b**) P25 after milling with melamine. (**c**) HR-TEM images of (**c**) P25 before milling and (**d**) P25 after milling with melamine. Red line is a guide for eye to observe a grain boundary.

Figure 1b,c show photographs of TiO_2_ particles obtained by milling without and with melamine, respectively. Ball milling of TiO_2_ with melamine produces a bright yellow-orange powder. Figure 2a,b show the TEM images of unmilled TiO_2_ and milled TiO_2_ with melamine, respectively, and those of HR-TEM images are shown in Fig. 2c,d. Both HR-TEM images show a lattice fringe of TiO_2_ crystal, and TEM images also indicate TiO_2_ nanoparticles with the size of a few tens of nanometers. Note that the TiO_2_ particles prepared by milling with melamine give aggregations, whose size become a few hundred of nanometers. The particle size after milling with melamine was also measured using DLS and was obtained 200 nm (Fig. S1), whose value is nearly equal to that of aggregated TiO_2_ particles observed by TEM, as shown in Fig. 2b. We also measured specific surface area of the TiO_2_ particles using BET adsorption/desorption measurements (Fig. S2). The data reveals that surface areas of the TiO_2_ particles before milling and after milling with melamine are 55.2 m^2^ g^−1^ and 15. 8 m^2^ g^−1^, respectively. This indicates that the surface area of TiO_2_ particles milled with melamine becomes smaller to 1/3, whose trend is consistent with TEM image, because aggregated particles reduce surface area.

**Figure 3:**
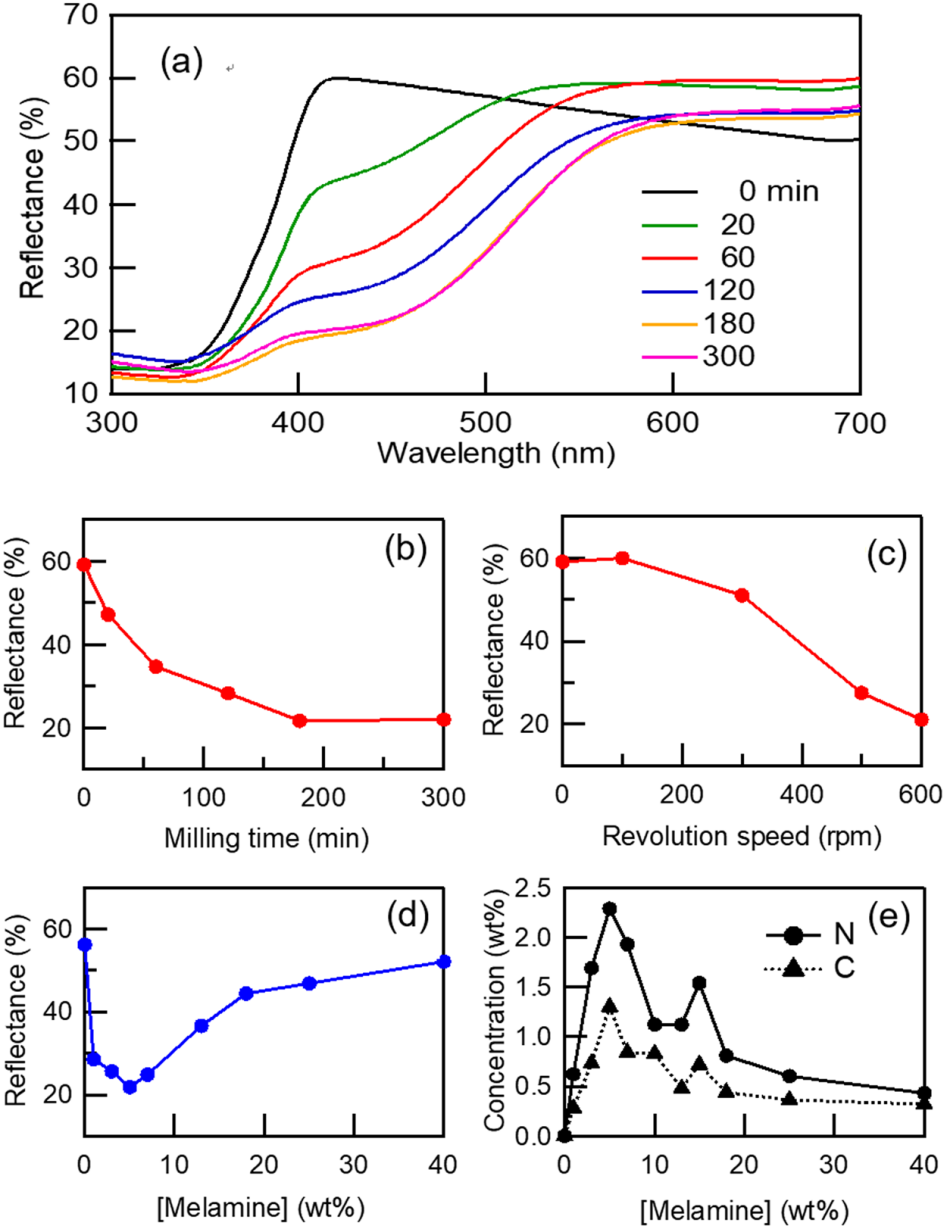
(**a**) Diffuse reflectance spectra of TiO_2_ milled with melamine as a function of milling time. Reflectance for TiO_2_ milled with melamine as a function of (**b**) milling time, (**c**) revolution speed (Fig. S3a), and (**d**) melamine concentration (Fig. S3b), measured at 450 nm. (**e**) N and C concentrations in TiO_2_ as a function of the melamine concentration.

Figure 3a shows diffuse reflection spectra of the TiO_2_ particles milled with melamine as a function of milling time, where the melamine concentration and revolution speed were set to 5 wt% and 500 rpm, respectively. The decreasing reflectance at around 400–600 nm is due to the absorption that results from the yellow-orange color. The same spectral profile has been typically observed in reflection and absorption spectra of either N-doped^15,31,39^ or N and C co-doped TiO_2_^40^. This absorption has been attributed to the electronic transition from a midgap level, composed of mixed N 2p and O 2p orbitals above the valence band, as observed using XPS^9,39,41^. Similar features were observed in the XPS spectra of the present study (*vide infra*).

Figure 3b,c show the reflectance measured at 450 nm as a function of the milling time (with the revolution set at 500 rpm, Fig. 3a) and the revolution speed (with the milling time set at 120 min, Fig. S3a), respectively, where the melamine concentration was set at 5 wt%. The reflectance decreased with an increase in the milling time or the revolution speed. Therefore, either longer milling times or faster revolution speeds provide higher absorption in the visible region.

Figure 3d,e show the reflectance (Fig. S3b) measured at 450 nm as a function of the melamine concentration and the corresponding dopant concentrations ([N] and [C]), respectively, using samples prepared at 500 rpm for 120 min. The reflectance of milled TiO_2_ initially decreased with increasing melamine concentration, but then increased at over 5 wt% melamine (Fig. 3d). A similar result is observed in Fig. 3e, where [N] and [C] increase with the melamine concentration, but decrease at over 5 wt% melamine. These trends indicate the most appropriate and efficient doping reaction is at 5 wt% melamine in TiO_2_. Large amounts of melamine act as a shock absorber during milling because the vessel and balls are hard ZrO_2_, whereas melamine is a soft organic material. Non-reacted melamine was physisorbed onto TiO_2_ at high melamine concentrations (15 wt%, Fig. S4), which were ensured to be removed by washing with four different solvents (see supporting info), i.e. elements before and after these washings were evaluated by CHN analysis (Table S1). Thus, the dopant concentrations of doped TiO_2_ with 5 wt% melamine were measured to be [N] = 2.3 wt% and [C] = 1.3 wt%, which are the highest concentrations reported to date, i.e. [N] = 1.2 wt% in a previous study^42^.

**Figure 4:**
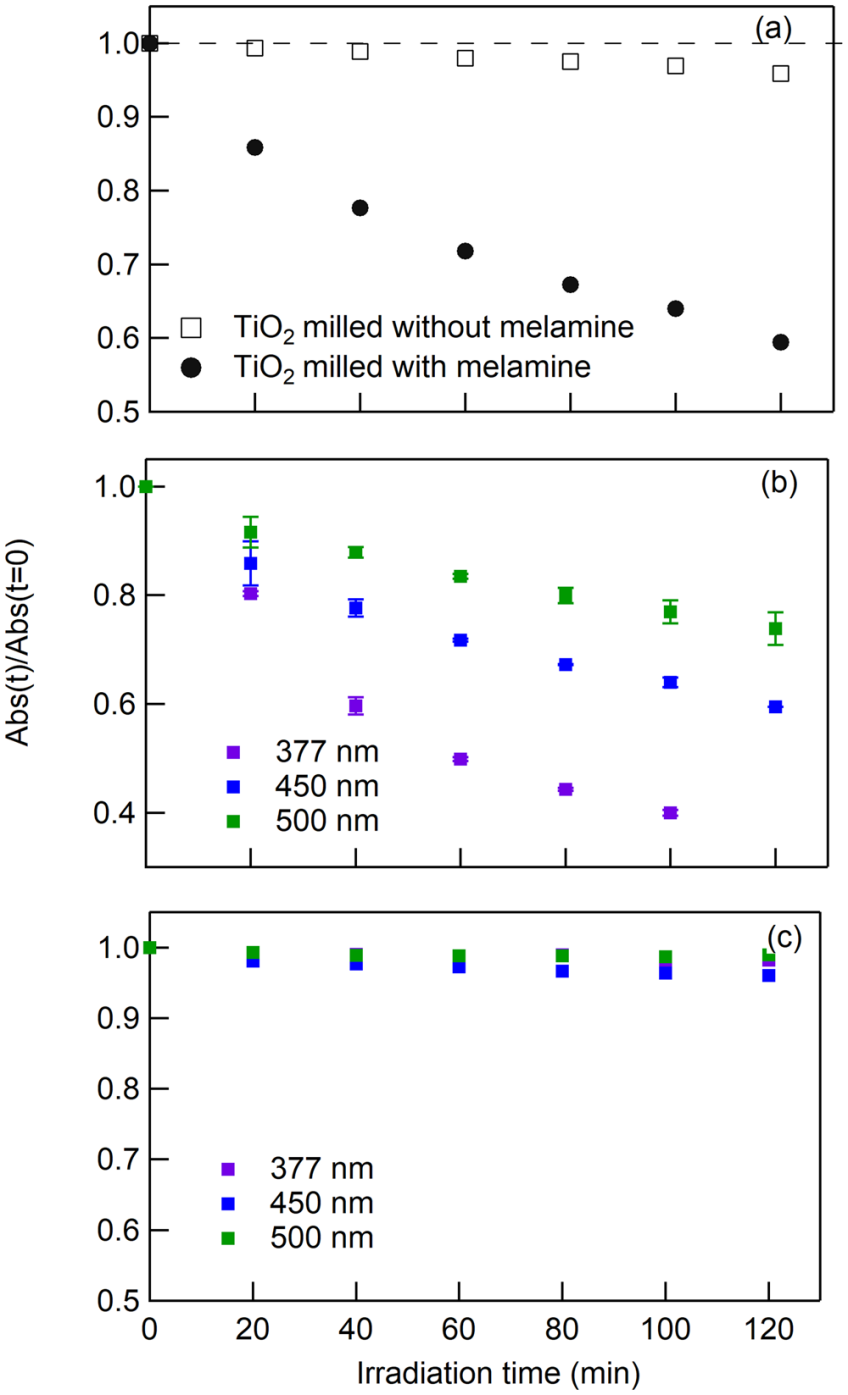
Absorbance of MB solution measured at 664 nm as a function of light irradiation time. (**a**) TiO_2_ milled with and without melamine. The wavelength of irradiated light is 450 nm. Absorbance of MB solution for different excitation wavelengths (**b**) with TiO_2_ and (**c**) without TiO_2_. The TiO_2_ photocatalyst was prepared by milling TiO_2_ at 120 min and 500 rpm with melamine (5 wt%) and without melamine.

The photocatalytic activity of milled TiO_2_ was evaluated by the photodegradation of MB, which was tracked by measurement of the absorption spectra (Fig. S5). The MB absorbance with TiO_2_, prepared by milling with and without melamine, was investigated by irradiation with visible light (λ_ex_ = 450 nm), and the results are shown in Fig. 4a. The absorbance decreases in the presence of TiO_2_ milled with melamine (●), whereas it does not change in the presence of TiO_2_ milled without melamine (□). The time profile of absorbance was investigated by variation of the excitation wavelengths (377, 450, and 500 nm), as shown in Fig. 4b. UV and visible wavelengths causes the degradation of MB in the presence of TiO_2_ milled with melamine. Figure 4c shows a time profile of the absorbance without TiO_2_. The profiles measured at all wavelengths did not change upon light irradiation; therefore, the self-decomposition of MB is excluded. In addition, no absorbance change as a function of irradiation time was confirmed under dark conditions (Fig. S6). Based on these results in Fig. 4a–c and Fig. S6, TiO_2_ milled with melamine exhibits photocatalytic activity under UV and visible light irradiation.

**Figure 5:**
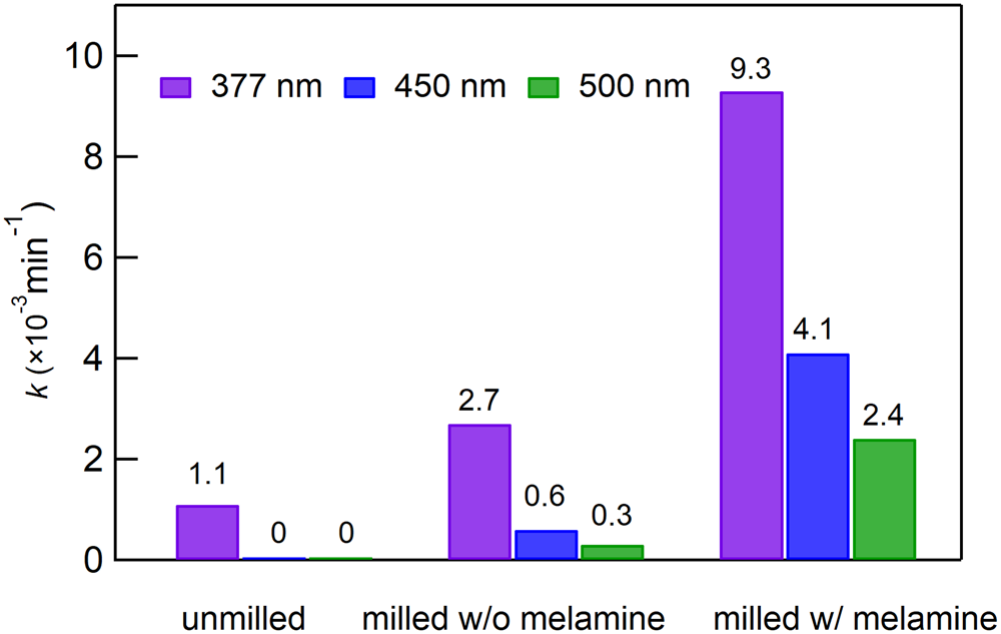
Rate constants, *k*, of photocatalytic reaction of MB. Used photocatalysts are unmilled TiO_2_(P25), TiO_2_ (P25) milled witout melamine, and TiO_2_ (P25) milled with melamine. Purple, blue, and green denote data irradiated at wavelengths of 377, 450, and 500 nm.

To quantify the photocatalytic activity, the time profiles in Figs 4b and S6 were evaluated with a single exponential function to obtain the rate constant *k* (min^−1^). Here, we carefully analyzed the time profiles of visible and UV light under the accurate conditions, i.e. same amount of TiO_2_, same spectral width of irradiation light, and irradiation lights of three wavelengths were set to the same intensities. In fact, note that quantitative comparison for photocatalytic activities of colored TiO_2_ in UV and visible light has not been reported in the previous studies under the same light intensity and monochromatic light. Figure 5 shows *k* for three samples (unmilled P25, and P25 milled with and without melamine) at the three wavelengths (377, 450, 500 nm). In all cases, *k* increased after milling. In particular, *k* measured at 450 and 500 nm were significantly enhanced, and were respectively 4 and 2 times higher than that before milling under UV irradiation at the same light intensity. Note that the activities of TiO_2_ prepared by present study clearly shows higher photocatalytic activity under visible lights than that of pristine one under UV light. Furthermore, the activities under the visible lights were, respectively, considered as 9 and 5 times higher than the UV under the solar light, according to the light intensities (see Fig. S7). As another important issue in the present study, the evaluation of the actives at the different wavelengths were accurately conducted at all the same experimental conditions, i,e, same amounts of TiO_2_ catalyst, same spectral widths of UV and visible lights, and same light intensities.

Here, we discuss several differences of the TiO_2_ particles before and after milling to further investigate high catalytic activity. According to the results of BET measurements, specific surface area of TiO_2_ powder was reduced to one third after milling with melamine, whose results are good agreement with the particle size of TEM measurements. In general, the reduced surface should result in the decrease of photocatalytic activity, because the reaction site is also decreased. Next, the crystalline phases of TiO_2_ milled with melamine were analyzed using XRD, and the results are shown in Fig. S8. The crystal structure of N and C co-doped TiO_2_ was similar to that of TiO_2_ milled without melamine, although there were several differences. Diffraction peaks emerged at 2θ = 32°, 44°, and 66° for TiO_2_ milled with melamine, which are attributed to the high-pressure α-PbO_2_ phase (also referred to as either srilankite or TiO_2_-II phase)^26–28,43–46^. Large amounts of TiO_2_ with the α-PbO_2_ phase were previously observed after high-energy milling of anatase TiO_2_, which was also considered to be responsible for the enhancement of photocatalytic activity^35,46^. In addition, it has been confirmed that the polymorphism of TiO_2_, such as rutile/anatase^18,47^ or anatase/brookite from experimental^31^ and theoretical studies^48,49^, results in higher photocatalytic activity than their pristine compositions, because the polymorphism increases the efficiency of electron-hole separation. Based on these previous studies^31,47–49^ and the present experimental results, it can be considered that the polymorphism observed in the current system, i.e., rutile, anatase, α-PbO_2_, and amorphous phases, has a significant effect on the enhancement of the photocatalytic activity.

**Figure 6:**
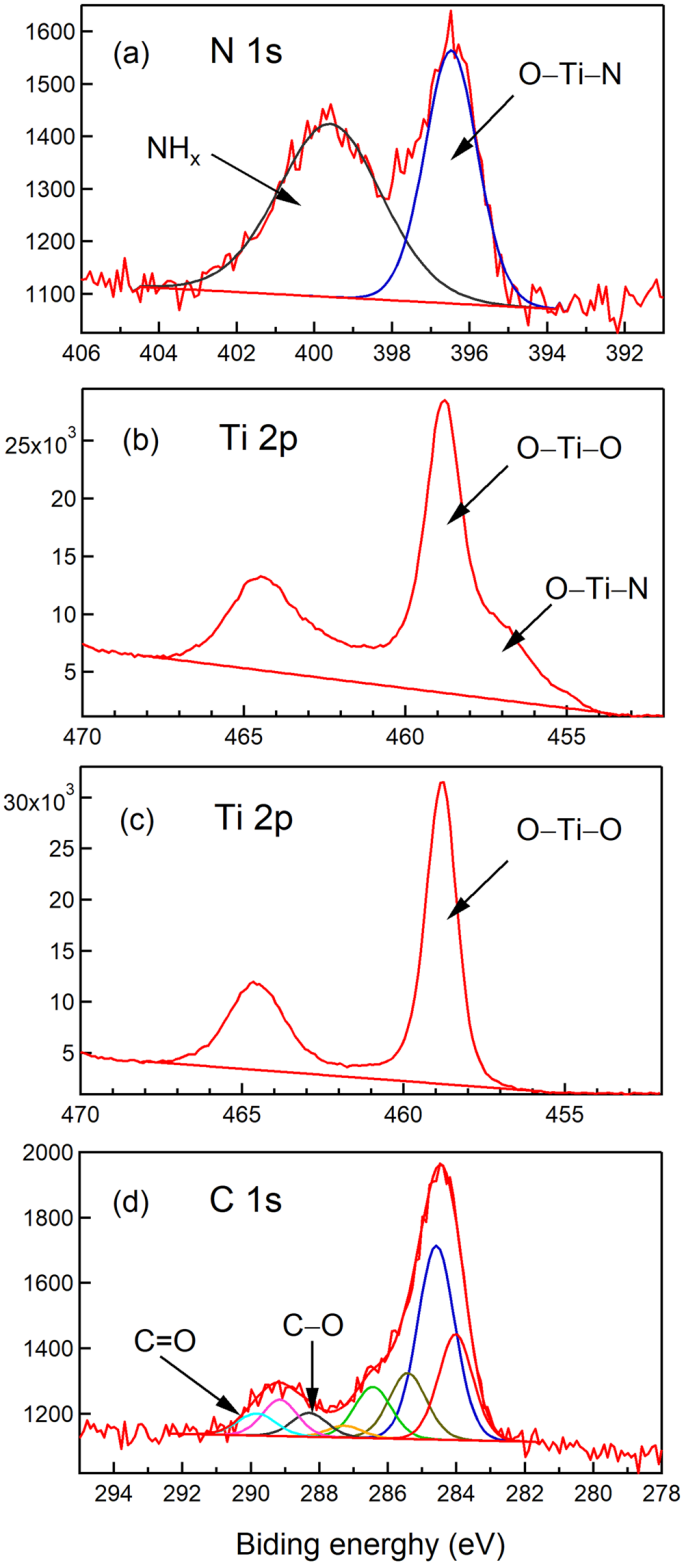
XPS spectra. (**a**) N 1s, (**b**) Ti 2p, (**d**) C 1s bands for TiO_2_ (P25) milled with melamine. (**c**) Ti 2p band for unmilled TiO_2_ (P25).

**Figure 7:**
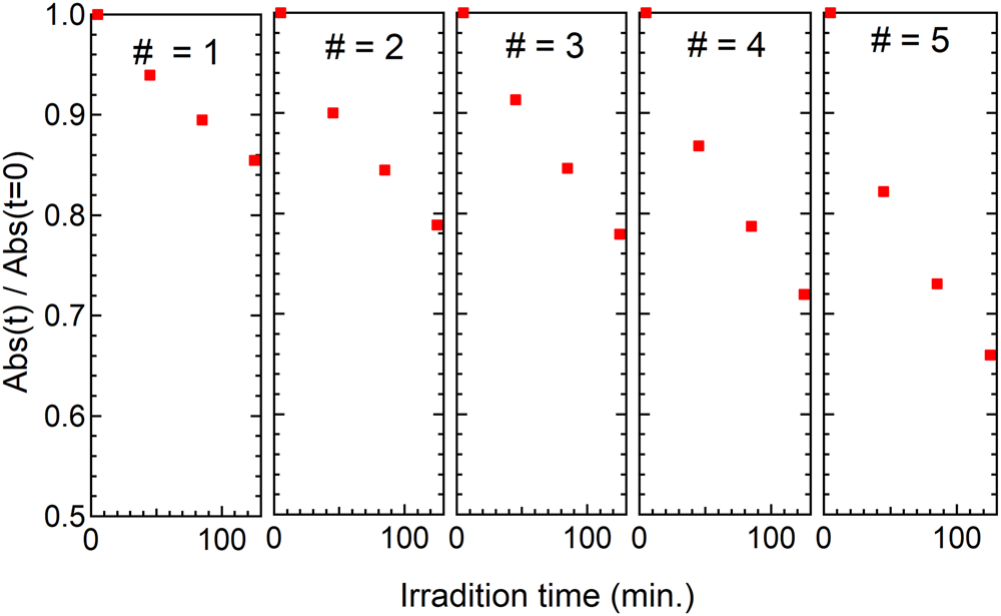
Cycle properties of TiO_2_ milled with melamine for MB photodecomposition reaction. This TiO_2_ photocatalyst was prepared by the ball milling for 120 min. at 500 rpm with the concentration of melamine of 5 wt%.

Finally, the electronic structure of TiO_2_ (P25) milled with melamine was measured using XPS (*vide supra*). Figure 6a shows the N 1 s spectrum, where the binding energy at 396.5 eV was attributed to an O-Ti-N bond. This reveals the substitutional replacement of an oxygen with a nitrogen atom (O-Ti-O → O-Ti-N) in the TiO_2_ lattice^8^. Fig. 6b,c show Ti 2p band of milled TiO_2_ and unmilled TiO_2_ with melamine, respectively. For the case of the band of milled TiO_2_ with melamine, the band Ti-N is observed at around 457 eV, which also ensures substitutional replacement of an oxygen with a nitrogen atom^8^. Thus, it was ensured that Ti-N bonding in TiO_2_ milled with melamine is observed by both the XPS spectra of N1s and Ti 2p. Figure 6d shows the C 1s spectrum. Although the band of Ti-C at 281.9 eV^50^ is not observed, the bands at 287.25 and 289.1 eV that have been attributed to C-O and C=O bonds, respectively, are observed^51,52^. This result suggests that the carbon dopants could be present as carbonate species in the TiO_2_ photocatalyst, prepared by ball milling with melamine. Lastly, let us mention a stability of the colored TiO_2_ photocatalyst prepared by the ball milling with melamine, briefly. We investigated cycle properties for the MB reaction using the same photocatalyst under visible irradiation upon the excitation wavelength of 450 nm. The sample was prepared by milling for 120 min at the revolusion speed of 500 rpm with the concetration of melamine of 5 wt%. The result is shown in Fig. 7. The data indicates TiO_2_ milled with melamine is used as a photocatalyst for repeated reactions. Namely, a good performance of the cycle was observed as the photocatalyst for the dye decomposition in an aqueous solution under the visible light irradiation to the colored TiO_2_ photocatalyst.

In summary, N and C co-doped TiO_2_ particles with a brilliant yellow-orange color were produced mechanochemically by high-energy ball milling. The particles integrated the N and C dopants up to 2.3 and 1.3 wt%, respectively, and the final particle size was 200 nm. The evaluation of the photocatalytic actives at the different wavelengths were accurately conducted at all the same experimental conditions using monochromatic light with the same intensities as well as the same amount of photocatalyst. Significant visible-light activity was observed. The synthesis process is very simple, fast, and environmentally benign, but produces a high-performance visible-light activated photocatalyst. In addition, large-scale synthesis could be established by the introduction of a large milling vessel.

## Methods

### Production of milled TiO_2_

A commercially available planetary ball milling apparatus (Premium line P-7, Fritsch Japan Co., Ltd.) was employed for high-energy ball milling and previously used for preparations of TiO_2_ nanopartices^35,53^. A ZrO_2_ milling vessel and ZrO_2_ milling balls were used to grind the TiO_2_ particles. The milling balls and TiO_2_ (1 g, P25, Degussa) were placed in the milling vessel. Milling was performed for various milling times (0 to 300 min), revolution speeds (0 to 600 rpm; revolution speed/rotation speed = −1:2), and melamine concentrations (0 to 40 wt%). The milling time experiment was conducted at 500 rpm for 30 min and with a melamine concentration of 5 wt%, followed by a pause time for cooling. This cycle was repeated to establish net milling times in the range of 0–300 min. The revolution speed experiment was conducted at a constant milling time of 120 min with a melamine concentration of 5 wt% at revolution speeds in the range of 0–600 rpm. The melamine concentration experiment was conducted at 500 rpm for 120 minutes with the melamine concentration varied in the range of 0–40 wt%. In the present study, no solvents were used for any of the milling procedures.

### Evaluation of milled TiO_2_

Transmission electron microscopy (TEM) and high-resolution transmission electron microscopy (HR-TEM) images were captured by a commercial instrument (JEM-2010, JEOL) at the condition of acceleration voltage of 200 kV. Samples of TEM measurements were prepared by dropping methanol solution of TiO_2_ particles onto the copper grid (EMJAPAN, U1017–5NM) and dried at ambient temperature over night. Dynamic light scattering (DLS; Zetasizer Nano, Malvern Instruments Ltd.) was used to determine the particle size of TiO_2_ dispersed in water. Diffuse reflection spectra were measured using a UV–Vis spectrophotometer (V-660, Jasco) equipped with an integrated sphere. The TiO_2_ powder was filled into a sample cell specilzied for poweder sample. To obtain the reflection spectrum, the reference spectrum was measured with a standard white plate. The nitrogen and carbon concentrations in TiO_2_ were measured by elemental analysis using a combustion CHN method (CHNS/0 2400II, PerkinElmer). Surface areas of TiO_2_ particles were measured by measuring adsorption and desorption profiles of N_2_ gas at 77 K with a commercial instrument (Belsorp-mini, Micro track Bell). The sample for BET measurements were heated at 150 °C for 3 hours before measurements. X-ray diffraction (XRD) was measured by a commercial instrument (Rint 2500, Rigaku) at the condition of Cu Kα, 40 kV, and 200 mA, and a sample-holder plate of nonreflective silicon specialized for producing a background-free signal was used. X-ray photoelectron microscopy (XPS) was measured by a commercial instrument (Kratos Nova, Shimadzu) at the condition of Al Ka monochromator.

### Evaluation of photocatalytic activity

Methylene blue (MB; Sigma-Aldrich) was used to evaluate the photocatalytic performance of TiO_2_. 3.0 mL of MB aqueous solution with concentration of 2.94 × 10^−5^ M, 1 mg of TiO_2_ particles, and a stirring bar were placed in a quartz cuvette with the optical path length of 1 cm. The photodecomposition reaction was performed at room temperature with light irradiated onto the cuvette while stirring the solution. As the light source of the photodecomposition reaction, the light from a Xe lamp was monochromated with a band-pass filter of 377, 450, and 500 nm after passing through an IR-cut filter. The spectral widths of the band-pass filters were 10 nm. The power of the 377, 450, and 500 nm light were set as 20 mW using a power meter in front of the cuvette. The irradiation time ranged from 0 to 300 min. During irradiation, the cuvette was enclosed by a black box to eliminate room lighting and stray light. After irradiation, the absorption spectra were recorded with a UV–vis spectrophotometer (V-660, Jasco). The spectra were collected using the transmission configuration with an integrated sphere to remove light scattering due to the TiO_2_ powder, which affects the absorbance. Time profiles of the absorption spectra were evaluated using the absorbance measured at a wavelength of 664 nm. The profiles were fitted using a single exponential function, i.e., *Abs*(*t*)/*Abs*(*t* = 0) = *a* + *b* exp(−*kt*), where *a*, *b*, and *k* are baseline, amplitude, and rate constant, respectively. According to the data as shown in Fig. S5a, the absorbance of MB solution with TiO_2_ particles under dark does not change. This indicates no change of absorption due to the adsorption of MB onto TiO_2_ particles. Figure 4c shows negligible changes of absorbance of MB solution without TiO_2_ particles under light irradiations at the three wavelengths. This indicates that a self-decomposition of MB by light irradiation is negligible at the excitations of three wavelength. Therefore, the above equation, *Abs*(*t*)/*Abs*(*t* = 0) = *a* + *b* exp(−*kt*), can extract the photocatalytic activity as kinetics data from the time profiles of absorbance as a function of time.

### Washing procedures of TiO_2_ particles

To evaluate the nitrogen and carbon concentrations, it is important to determine whether or not non-reactive melamine is physisorbed onto the milled TiO_2_ particles. The physical adsorption of melamine onto TiO_2_ influence the measured concentrations of nitrogen and carbon doped in TiO_2_. Therefore, the nitrogen and carbon concentrations were carefully measured before and after washing the milled TiO_2_, because melamine is high water-soluble. The washing process involved dissolving the milled TiO_2_ (70 mg) in water (10 mL) and heating to 70 °C with stirring for 10 min. The solution was then filtered and the obtained TiO_2_ powder was dried at 90 °C for 30 min. The nitrogen and carbon concentrations were measured before and after the washing process by CHN elemental analysis. The nitrogen and carbon concentrations decreased after the washing process when the TiO_2_ was milled with melamine at concentrations greater than 5 wt% (Fig. S4). In contrast, the nitrogen and carbon concentrations did not change after washing for melamine concentrations lower than 5 wt%. Therefore, it was determined that higher melamine concentrations required the washing process prior to nitrogen and carbon measurements. In addition, the washing process was conducted using other solvents (acetone, cyclohexane, CHCl_3_, and CCl_4_) to determine if other products such as water-insoluble were attached to TiO_2_. TiO_2_ prepared by milling with 10 wt% melamine concentration after the hot water washing process was used. The nitrogen and carbon concentration did not change by washing with organicsolvents, as shown in Table S1.

## Electronic supplementary material

Supplementary Information
