# Supplementary material for: Mechano-synthesized orange TiO2 shows significant photocatalysis under visible light
Source: Sci Rep. 2018 Oct 19;8:15549. doi: 10.1038/s41598-018-33772-6 (PMC6195626; doi:10.1038/s41598-018-33772-6)
Supplement: Supplementary file 1 — Supplementary Information [file 41598_2018_33772_MOESM1_ESM.docx]

**Supporting Information**

**Mechano-synthesized orange TiO_2_ shows significant photocatalysis under visible light**

Ken-ichi Saitow^*a,b^, Yufeng Wang^b^, and Shintaro Takahashi^b^

^a^ *Natural science center for Basic R&D (N-BARD), Hiroshima University,*

*1-3-1 Kagamiyama, Higashi Hiroshima, Hiroshima 739 8526, JAPAN.*

^b^ *Department of chemistry, Graduate school of science, Hiroshima University,*

*1-3-1 Kagamiyama, Higashi Hiroshima, Hiroshima 739 8526, JAPAN.*

**Analysis of crystal structures by X-Ray Diffraction (XRD)**

The crystal structure of N and C co-doped TiO_2_ (Fig. S8b) was similar to that of TiO_2_ milled without melamine (Fig. S8a), both of which showed broader peaks than the pristine TiO_2_ (Fig. S8d), indicated this mechanochemical process generated considerable number of amorphous phase. But significant diffraction peaks emerge at 2θ = 32°, 44°, and 66° in the TiO_2_ milled with melamine, which are attributed to the high pressure phases, called as either α-PbO_2_, TiO_2_-II, or srilankite phases.

An XRD pattern for TiO_2_ milled with a high melamine concentration (40%) was also measured; however, the diffraction pattern did not change after milling and was with the same as that of the unmilled TiO_2_ (P25). This insufficient milling is considered to be due to the large amounts of melamine that act as a shock absorber during milling.

We also conducted XRD measurements before and after the reaction as a function of reaction cycles. The data shows no change after long-time reaction, as displayed in Fig. S9.

**Elemental analysis by X-Ray photoelectron spectroscopy (XPS)**

We measured the XPS spectrum for obtaining the atomic ratios. According to the data in Fig. S10 and table S1, the principle element observed at the surface of TiO_2_ is Ti and O before and after milling. N is a minor component and its atomic ratio is in good agreement with that obtained from CHN analysis, indicating bulk analysis. As for carbon, it is a 2^nd^ minor component. In addition, the atomic ratio of carbon after milling with melamine is the same to that of pristine P25 before milling. Therefore, it was considered that the carbon at surface measured by XPS is not due to melamine but an intrinsic adsorbate at the surface of P25.

Figure S1. Particle size distribution of milled TiO_2_ particles dispersed in water. (a) Time correlation function of DLS measurement and (b) size distribution of TiO_2_ particles obtained from the time correlation function. The sample was prepared by milling at 500 rpm for 2 h with 5 wt% melamine.

(b)

(a)

(d)

(c)

Figure S2. Adsorption and desorption curves of (a) unmilled P25 and (b) P25 milled with 5 wt% melamine. BET plots of (c) unmilled P25 and (d) P25 milled with 5 wt% melamine. Surfaces areas of unmilled P25 and P25 milled with melamine are obtained as 55.2 and 15.8 m^2^g^-1^, respectively.

Figure S3. Diffuse reflection spectra for TiO_2_ particles milled with melamine. (a) TiO_2_ prepared at various revolution speeds for 120 min with 5 wt% melamine. (b) TiO_2_ prepared with various melamine concentrations milled at 500 rpm for 120 min.

Figure S4. Nitrogen and carbon concentrations in milled TiO_2_ as a function of melamine concentration. Nitrogen and carbon concentrations were obtained by CHN elemental analysis before and after washing with hot water. Milling was conducted at 500 rpm for 120 min.

**Table S1** Nitrogen and carbon concentrations measured by CHN elemental analysis via various washing processes.

(a)

(b)

Figure S5. Time evolution of MB absorption spectra by irradiation with an Xe lamp. Spectra of the irradiated MB solution containing (a) TiO_2_ milled without melamine (500 rpm and 120 min.) and (b) TiO_2_ milled with 5 wt% melamine (500 rpm, 120 min). The light source for the data of (a) and (b) is the Xe lamp without band pass filters. (c) TiO_2_ milled without melamine (500 rpm and 120 min.) and (d) TiO_2_ milled with 5 wt% melamine (500 rpm, 120 min). The light source for the data of (c) and (d) is a monochromated light (450 nm) from the Xe lamp with a band pass filter and IR cut filter.

(d)

(c)

Fig. S6 (a) The absorbance change of MB in the dark with N,C co-doped TiO_2_ samples. (b) The absorbance change of MB with TiO_2_ milled without melamine. (c) The absorbance change of MB with pristine TiO_2_ (P25) as purchased.

(b)

(a)

(c)

Dark w TiO_2_

Figure S7. Power spectrum of the solar light (AM1.5) at the global tilt condition. The power spectrum data is from ref. 1. The average power obtained within three wavelength regions of 377±5, 450±5, 500±5 nm are indicated by purple, blue, and green dashed lines, respectively.

Figure S8. XRD patterns of TiO_2_ milled (a) without melamine, (b) with 5 wt% melamine, and (c) with 40 wt% melamine. Milling was conducted for 120 min and at 500 rpm. XRD pattern of (d) as-received TiO_2_ (no milling).

Figure S9. XRD diffractions of TiO_2_ milled with 5 wt% melamine as a function of the number of cycles for the MB photodecomposition reaction. The diffraction patters do not change as the number of cycles increases. Milling was conducted for 120 min and at 500 rpm.

**
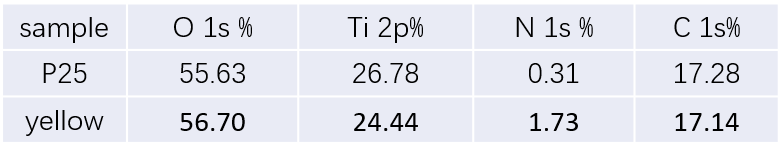
**

O 1s

C 1s

O KLL

Ti LMM

Ti 2p

N 1s

Ti 3s

Ti 3p

Figure S10. XPS spectrum to evaluate element of TiO_2_ sample. The results of atomic ratio are listed in the above table.
